# Melancholia

**Published:** 1894-06

**Authors:** 


MELANCHOLIA.—On the subject of Melancholia Spitzka's work on insanity, says: The duration of melancholia may comprise weeks, months, or years. The average duration is from three to eight months. The frequency is difficult to determine, as a large number of the patients never reach the asylum, but recover, die, or commit suicide outside its walls. Of 2,297 admissions to the pauper insane asylum for males in New York City, 301, or a little over thirteen per cent., were cases of melancholia. Of 1,193 patients of both sexes admitted to the reception wards of Professor Meynert, not quite six per cent. were melancholiacs. The greater frequency of melancholia with females, which is in part attributed to the influence of prolonged lactation, pregnancy, exhausting discharges, and chloro-anemia in provoking this psychosis, is illustrated by the statistics of the Buda-Pesth asylum. Here ten per cent. of the male and seventeen per cent. of the female patients suffered from melancholia. Of 146 males received at Feldhof by Krafft-Ebing, 13, or nearly seven per cent., were melancholiacs, while of 121 females, 18, or nearly fifteen per cent., suffered from the same disease.

In the writer's statistics the striking fact is noticeable that melancholia is most frequent in the Teutonic people, the ones who, according to Morselli, also show the highest suicidal ratio; and among these it was found more frequent with those who had immigrated than with those who had been born in this country.—*Goldberg, N. Y., Medical Journal.*

